# Colchicine in Coronary Artery Disease—Too Much of a Good Thing?

**DOI:** 10.3390/jcdd13060284

**Published:** 2026-06-22

**Authors:** Hui Zhen Lo, Sanjay Patel, Jamie Layland

**Affiliations:** 1 Bayside Health (Peninsula), Frankston, VIC 3199, Australia; 2 Department of Cardiology, Royal Prince Alfred Hospital, Camperdown, NSW 2050, Australia; 3 Coronary Diseases Group, Heart Research Institute, Newtown, NSW 2042, Australia; 4 Department of Cardiology, Bayside Health (Peninsula), Frankston, VIC 3199, Australia; 5 Department of Medicine, Peninsula Clinical School, Monash University, Frankston, VIC 3199, Australia

**Keywords:** colchicine, coronary artery disease

## Abstract

Coronary artery disease (CAD) is the leading cause of mortality and morbidity in the world. While low-density lipoprotein cholesterol has been the main target of secondary prevention, inflammation has gained traction as a potential target for reducing adverse cardiovascular events. Hence, in this review, we aim to outline the current evidence base for the use of colchicine in CAD to provide more clarity from the findings in the recent trials and meta-analyses (LoDoCo2, COLCOT, COPS, CONVINCE, CLEAR-SYNERGY, COLOCT, and EKSTROM). Given colchicine’s low cost and widespread availability, it is a potential adjunct to lipid-lowering therapy. However, it may not be as effective in the secondary prevention of CAD as previously thought. Ongoing research on colchicine remains vital to determine its utility in patient populations beyond those with CAD as well as to better understand the fine balance between its therapeutic benefits and potential side effects.

## 1. Introduction

Although there have been notable advancements in recent decades, cardiovascular disease (CVD) continues to be the leading cause of morbidity and mortality globally [1]. Currently, the treatment for CAD consists of non-pharmacological and pharmacological management. The traditional cardiovascular risk factor reduction strategies include a healthy diet, physical activity, and cessation of alcohol and smoking. Blood pressure control, antiplatelet therapy, and lipid-lowering therapies—high-dose statins, ezetimibe, and PCSK9 inhibitors—are key to managing this pathological process [2].

Targeting low-density lipoprotein cholesterol (LDL-C) has become a central component of secondary preventive care, and addressing inflammation has also emerged as a potential method for reducing the risk of cardiovascular events [3]. The Canakinumab Anti-inflammatory Thrombosis Outcome Study (CANTOS) provided initial evidence that targeting inflammation may improve cardiovascular outcomes [4]. However, the high cost of canakinumab along with the increased rate of fatal infections made it a less promising drug [4]. Colchicine, on the other hand, is inexpensive, safe, and widely available.

Colchicine received further attention after the LoDoCo2 and COLCOT trials reported favourable results, prompting both the ESC and AHA to support its use for secondary prevention [2,5,6,7]. However, subsequent findings from the CONVINCE and CLEAR-SYNERGY trials have moderated earlier expectations regarding colchicine [8,9]. Given the increased number of trials and meta-analyses on this topic, we aim to evaluate current evidence to provide an up-to-date review on the utility of colchicine in coronary artery disease.

## 2. Chronic Inflammation in Atherosclerosis

Atherosclerosis is a chronic, progressive disease centred around inflammation as well as the innate and adaptive immune response [10]. The process is initiated when endothelial dysfunction results in LDL infiltration and accumulation in the tunica intima. The LDL particles oxidise, which triggers activation and production of inflammatory mediators that recruit circulating monocytes. These monocytes then differentiate into macrophages, which absorb oxidised LDLs, in turn becoming foam cells. Subsequently, vascular smooth muscle cells migrate to the area, which helps in the development of the fibrous plaque with a necrotic core and fibrous cap [11]. As atherosclerotic plaque grows, it can become unstable, subsequently resulting in plaque rupture and major cardiovascular events (MACE).

This chronic low-grade inflammation in patients has been proven to be a strong independent factor of MACE despite being on secondary prevention, as assessed by raised high-sensitivity CRP (hsCRP) levels [12]. The PROVE IT-TIMI 22 trial demonstrated that patients with lower CRP levels after statin therapy had better clinical outcomes compared to those with higher CRP levels, and this was independent of LDL-C levels [13].

The NLR family pyrin domain containing 3 (NLRP3) inflammasome is central to the residual inflammatory risk hypothesis. In atherosclerosis, cholesterol deposits, oxidised LDLs and danger-associated molecular patterns (DAMPs) are the main triggers for NLRP3 activation [14]. Upon activation, it triggers the secretion of cytokines like interleukin-1β (IL-1β), interleukin 6 (IL-6), and interleukin-18 (IL-18) [11]. The result is an increased inflammatory response and oxidative stress in endothelial cells. This persistent activation of NLRP3 and the interleukin cascade continues as an ongoing feedback loop. Specifically, IL-6 plays a key role in stimulating the production of acute-phase reactants like hsCRP, which further drives the recruitment and activation of inflammatory cells [15].

## 3. Mechanism of Action of Colchicine

**Figure 1:**
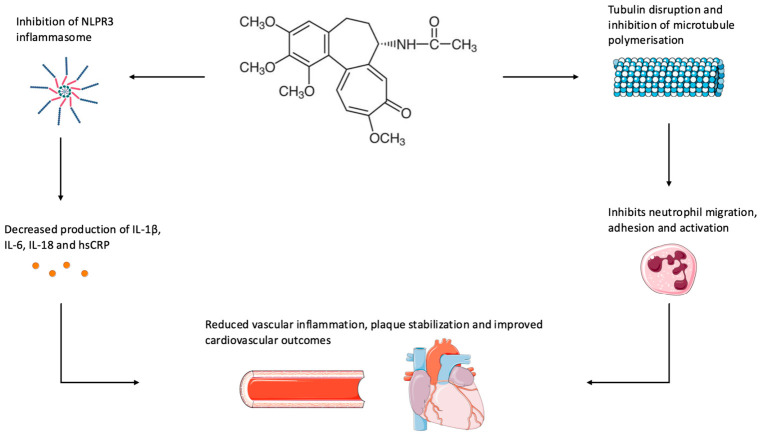
Mechanism of action of colchicine.

Colchicine is an oral medication that binds to tubulin, inhibiting tubulin polymerisation and microtubule formation [16,17] (Figure 1). This disrupts downstream cellular functions, including the cellular cytoskeleton, mitosis, and intracellular protein transport. Consequently, it leads to slower plaque growth, plaque stability, and prevention of plaque rupture with the suppression of secretion of smooth muscle cells, myofibroblasts, and fibrosis [18]. Colchicine also results in diminished surface expression of L-selectin adhesion molecules and E-selectin adhesion molecules and downregulates tumour necrosis factor receptors on macrophages and endothelial cells [18]. This inhibits neutrophil migration, adhesion, and activation, as well as leukocyte adhesion to the endothelium. Most importantly, colchicine has the ability to suppress the NLRP3 inflammasome. The NLRP3 inflammasome is present in myeloid cells and produces inflammatory cytokines IL-1β and IL-18 capable of promoting oxidisation of LDL, foam cell production, and progression of atherosclerotic plaques. Hence, inhibiting the NLRP3 inflammasome is helpful in preventing the activation of the innate immune response, preventing plaque progression and plaque rupture [16]. The NLRP3 inflammasome also triggers IL-6 production, which promotes hs-CRP production [16]. The use of colchicine also has downstream effects of reducing hs-CRP.

## 4. Trials and Clinical Utility

Clinical characteristics of each study were extracted and presented in Table 1. We included data on population, colchicine dose, comparator, follow-up duration, baseline hsCRP and primary outcomes of studies.

### 4.1. Low-Dose Colchicine (LoDoCo2) Randomised Controlled Trial

The LoDoCo2 trial was a double-blinded randomised controlled trial that was conducted in Western Australia and the Netherlands [5]. A total of 5522 patients with stable coronary artery disease were enrolled, identified by invasive coronary angiography, computed tomography angiography, or a coronary-artery calcium score of 400 Agatston units or higher. Notably, 84% of these participants had a history of acute coronary syndrome (ACS).

Prior to trial initiation, there was a lead-in period of one month where patients received 0.5 mg of colchicine daily. This ensured only compliant patients without adverse side effects were included in the trial. Patients in the colchicine group continued to have 0.5 mg of colchicine daily with follow-ups every 6 months and a median follow-up duration of 28.6 months (IQR: 20.5–44.4). The LoDoCo2 trial demonstrated a significantly lower risk of cardiovascular death, spontaneous myocardial infarction, ischaemic stroke or ischaemia in the colchicine group compared to the placebo group (6.8% vs. 9.6%; HR: 0.69; 95% CI: 0.57–0.83; *p* < 0.001). Although insignificant, non-cardiovascular deaths and deaths from any cause had a higher incidence in the colchicine group compared to the placebo group, with a hazard ratio of 1.86 (0.99–3.48) and 1.21 (0.86–1.71), respectively. Unlike CANTOS, hsCRP was not routinely measured in LoDoCo2 and was not a prerequisite for randomisation [4].

### 4.2. Colchicine Cardiovascular Outcomes Trial (COLCOT)

The double-blinded COLCOT randomised a total of 4745 patients who had a myocardial infarction or planned percutaneous coronary interventions in the preceding 30 days. Patients in the colchicine group had 0.5 mg of colchicine daily, and all patients were followed up at the one-month mark and at every three months thereafter, with a median follow-up of 22.6 months. COLCOT demonstrated a significantly lower risk of cardiovascular death, resuscitated cardiac arrest, myocardial infarction, stroke and urgent hospitalisation for angina leading to coronary revascularisation in the colchicine group compared to the placebo group (5.5% vs. 7.1%; HR: 0.77; 95% CI: 0.64–0.96; *p* = 0.02) [6]. In addition, adverse events, including pneumonia, nausea and flatulence, were statistically significant in the colchicine group (0.09% vs. 0.4%, *p* = 0.03; 1.8% vs. 1.0%, *p* = 0.02; 0.6% vs. 0.2%, *p* = 0.02).

In COLCOT, only 207 patients had both baseline and post-treatment hsCRP measured (99 colchicine, 108 placebo). After six months, hsCRP dropped by 70% in the colchicine group and 66.6% in the placebo group, but the small sample size limits direct comparison. Notably, the median on-treatment hsCRP was 1.37 mg/L—well below the 2 mg/L threshold linked to outcomes in the CANTOS trial.

### 4.3. Colchicine in Patients with Acute Coronary Syndrome (COPS) Trial

The COPS trial was a double-blinded randomised controlled study conducted in Australia, enrolling 795 patients diagnosed with ACS and CAD [19]. CAD diagnosis was established via coronary angiography, defined as at least 30% luminal stenosis in an epicardial vessel measuring a minimum of 2.5 mm in diameter. Patients were treated either with percutaneous coronary intervention or received medical therapy. The colchicine regimen consisted of 0.5 mg administered twice daily for one month, followed by 0.5 mg once daily for the subsequent eleven months, with follow-up assessments at one, six, and twelve months. The study demonstrated a reduced incidence of the composite endpoint—including all-cause mortality, ACS, ischaemia-driven urgent revascularisation, and non-cardioembolic ischaemic stroke—at 12 months in the colchicine group compared with placebo (6.1% vs. 9.5%, HR: 0.65; 95% CI: 0.38–1.09; *p* = 0.09). However, rates of all-cause death and non-cardiovascular mortality were higher among patients receiving colchicine (2.0% vs. 0.25%, *p* = 0.018; 1.3% vs. 0%, *p* = 0.023). A subsequent meta-analysis including these three trials did not reveal any significant concerning signal for non-cardiovascular death [3].

### 4.4. CONVINCE Randomised Controlled Trial

The CONVINCE trial randomised 3144 patients with non-severe ischaemic stroke or high-risk transient ischaemic attack (TIA) within 72 h and 28 days to 0.5 mg colchicine daily or guideline-based therapy [8]. Patients with a modified Rankin Scale score of three or less were defined as having a non-severe ischaemic stroke, and they made up the bulk of patients in the trial (88.0% colchicine vs. 87.8% control). Patients who had transient focal motor or speech deficits, including an ABCD2 score of at least four, 50% or more stenosis of the large artery lumen, or hyperintensity on diffusion-weighted magnetic resonance imaging (MRI) with focal neurological deficits, were included as a high-risk TIA. These patients were followed up at 28 days, 90 days, and at six-month intervals thereafter with a median follow-up of 36 months. At 36 months of follow-up, the colchicine group had a non-significant lower incidence of first recurrent non-fatal ischaemic stroke, myocardial infarction, cardiac arrest, or hospitalisation in the colchicine group (9.8% vs. 11.7%; HR: 0.84; 95% CI: 0.68–1.05; *p* = 0.12).

In the trial, most patients had baseline hsCRP recorded, which showed a statistically significant decrease over three years (2.71 mg/L to 1.52 mg/L for colchicine and 2.82 mg/L to 1.91 mg/L for the control, *p* = 0.02). Although there was a significant reduction in hsCRP, 86.4% of patients had measurements at baseline compared to only 26.4% at the three-year mark. This notable decrease in sample size may affect the reliability of the results. One limitation of the trial was the absence of a placebo group, as the comparison group received guideline-based management only. The study also enrolled patients with high-risk TIA, a diagnosis that can be subjective due to the lack of definitive biomarkers or imaging criteria. Additionally, the trial occurred during the COVID-19 pandemic, leading to a two-month pause in recruitment, an extension of the overall trial duration by more than a year, and incomplete follow-up, factors that may have contributed to 8% fewer outcomes than anticipated and less conclusive results. During the pandemic, higher rates of stroke were observed among patients with COVID-19, as indicated by a retrospective cohort study in New York that reported a greater likelihood of stroke in individuals with COVID-19 compared to those with influenza infection (OR: 7.6, 95% CI: 2.3–25.2) [20]. The reduced enrolment rates during the pandemic may therefore be associated with patient hesitancy to attend hospitals during this period.

### 4.5. CLEAR-SYNERGY (OASIS 9) Trial

The most recent CLEAR-SYNERGY (OASIS 9) trial investigated patients with myocardial infarction (STEMI and large NSTEMI) who were enrolled within 72 h from their index PCI, and its findings were a stark contrast to those of LoDoCo2 and COLCOT [9]. A total of 7062 patients were included, and the majority of patients presented with a STEMI (95.3% colchicine vs. 94.8% placebo). In this trial, the dosage of colchicine was dependent on weight in the initial 90 days—0.5 mg twice a day for patients who were at least 70 kg and 0.5 mg once daily for patients less than 70 kg. All patients in the colchicine group had 0.5 mg once daily after the first 90 days. However, the protocol was changed during the study, and a reduced dose of colchicine was used (0.5 mg for all patients). After a median follow-up of three years, there was no significant reduction in the incidence of cardiovascular deaths, recurrent myocardial infarction, stroke, or unplanned ischaemia-driven coronary revascularisation (9.1% vs. 9.3%; HR: 0.99; 95% CI: 0.85–1.16). In addition, diarrhoea was a statistically significant adverse effect that occurred in the colchicine group (10.2% vs. 6.6%), which was not the case in COLCOT [5,6].

While hsCRP levels had a statistically significant least-squares mean level at three months (colchicine: 2.98 ± 0.19 mg/L vs. placebo: 4.27 ± 0.19 mg/L), inflammation was not controlled compared to levels seen in COLCOT and CANTOS [4]. In COLCOT, even though baseline hsCRP was high, colchicine still managed to result in a 70% reduction in hsCRP after six months from 4.27 mg/L to 1.37 mg/L [6].

This trial was conducted during the COVID-19 pandemic, which may have contributed to the low incidence of major adverse cardiovascular events (MACEs) reported. In a pre-pandemic analysis of death from cardiovascular causes, myocardial infarction, stroke, or revascularization, the hazard ratio was 0.78 (95% CI: 0.60–1.02), compared to 1.09 (95% CI: 0.88–1.35) during and 1.19 (95% CI: 0.79–1.78) after the pandemic. The pre-pandemic analysis indicated that colchicine might have had a favourable effect on MACE. During the pandemic, there was underreporting and fewer hospitalisations for non-fatal myocardial infarctions. Similar trends were observed in other trials conducted during the COVID-19 pandemic. A meta-analysis involving over 80,000 patients showed a significant reduction in the daily number of admissions for myocardial infarction during lockdown compared to the pre-pandemic period (14.7 vs. 40.6 patients per day) [21]. These factors likely influenced the findings of the CLEAR-SYNERGY trial as well as other studies conducted during the pandemic, such as IRONMAN and GUIDE-HF [22,23]. However, it is important to note that given the exploratory nature of this analysis, our viewpoints should be considered as hypothesis-generating rather than confirmatory.

Furthermore, in the spironolactone arm of the trial, the results were discordant with prior studies using mineralocorticoid receptor antagonists [24,25]. This raises further questions regarding the validity of the results obtained during the CLEAR-SYNERGY trial due to the effects of the COVID-19 pandemic.

Despite the findings from the CLEAR-SYNERGY trial, the updated systematic review and meta-analysis by Samuel et al., which includes the CLEAR-SYNERGY trial, still showed that colchicine resulted in a 25% reduction in MACE, a 29% reduction in myocardial infarction, a 37% reduction in ischaemic stroke and a 33% reduction in urgent coronary revascularisation [3]. This continues to support the ongoing use of colchicine as secondary prevention of cardiovascular events as per the American and European guidelines [2,7].

### 4.6. Colchicine–Optical Coherence Tomography Trial (COLOCT)

This double-blinded Colchicine–Optical Coherence Tomography Trial (COLOCT) included 104 patients who had been diagnosed and hospitalised with ACS within one month [26]. Patients had to have at least one non-culprit lesion with a stenosis diameter percentage of 30% to 70% estimated visually on coronary angiography and a lipid-rich plaque demonstrated on optical coherence tomography. Patients in the colchicine group had 0.5 mg of colchicine daily and were followed up at 12 months. COLOCT showed a statistically significant reduction in minimal fibrous cap thickness at the 12-month follow-up (87.2 μm; 95% CI: 69.9–104.5 μm vs. 51.9 μm; 95% CI: 32.8–71.0 μm). This supports the underlying mechanism of action of colchicine through plaque modification and stabilisation of plaque through inhibition of microtubule formation [27]. Though statistically insignificant, the colchicine group had lower incidences of major adverse cardiovascular and cerebrovascular events, defined as a composite of all-cause death, nonfatal myocardial infarction, nonfatal stroke, and revascularization because of ischemia (11.5% vs. 17.3%; *p* = 0.402).

### 4.7. EKSTROM Trial

The EKSTROM double-blinded randomised controlled trial randomised 72 patients with atherosclerosis proven on coronary angiography or computed tomography coronary angiography [28]. Patients in the colchicine group had 0.5 mg of colchicine daily and were followed up at three months, six months and 12 months. At 12 months, patients treated with colchicine had a significant reduction in total plaque volume compared to placebo. There was a statistically significant change in percent atheroma volume (PAV) of −1.1% (*p* = 0.009) between the colchicine and placebo groups. There was also a statistically significant reduction in dense calcified plaque volume of −0.9% PAV between the colchicine and placebo groups (*p* = 0.009). Prior studies have associated a 1% reduction in PAV with a 20% relative risk reduction in MACE [29]. Therefore, these results provide mechanistic support to colchicine studies performed pre-pandemic that showed a favourable outcome for patients with CVD treated with colchicine.

## 5. Safety Considerations

Colchicine has a narrow therapeutic index, with toxic doses at 0.1 mg/kg or more and potentially fatal doses at 0.8 mg/kg [30]. The most common adverse events are gastrointestinal, which include diarrhoea, vomiting and nausea. As noted in the CLEAR-SYNERGY and COLCOT trials, diarrhoea and nausea were significant adverse events in the colchicine group, respectively [6,9]. However, these gastrointestinal effects are often mild and can be avoided by starting with a low dose and slowly increasing to the therapeutic dose.

Colchicine has several drug interactions that need to be considered prior to commencement. Colchicine is a substrate for the efflux transporter P-glycoprotein and CYP3A4. Hence, it should be avoided in patients who are on CYP3A4 inhibitors (e.g., clarithromycin, itraconazole, ketoconazole, ritonavir) or G-glycoprotein inhibitors (e.g., cyclosporine, ranolazine) due to the risk of toxicity [31]. The concomitant use of colchicine and statin also increases the risk of myopathy since both medications are metabolised by P-glycoprotein and CYP3A4 [30].

Furthermore, prolonged use of colchicine is associated with bone marrow suppression. It is worth noting that the COLCOT did show that pneumonia was a significant adverse event in the colchicine group, and this may be related to reduced immunity in these patients [6]. A population-based cohort study by Tsai et al. also demonstrated a higher incidence of pneumonia in the colchicine group compared to the placebo group (18.6 vs. 126 per 1000 person-years, *p* < 0.05) [32]. As colchicine is metabolised in the liver and kidneys, it is important to consider dose adjustment in patients with renal or hepatic impairment.

## 6. Clinical Implications

The increase in the number of RCTs in the recent few years has been imperative in providing a large evidence base on the utility of colchicine in CAD. Early landmark trials like LoDoCo2 and COLCOT showed favourable results for colchicine in patients with CAD or MI compared to placebo. However, adverse events (pneumonia, nausea, flatulence) were significant in the colchicine group compared to the placebo group in COLCOT. Non-cardiovascular mortality was also significant in the colchicine group in COPS. However, in these trials, there is a skewed proportion of males compared to females, with approximately 20% or fewer women in the sample population, which is not to be expected in the general population. This is especially important given the differences in male and female physiology and the association with coronary artery disease. Pre-menopausal females have a protective factor for atherosclerosis and cardiovascular events due to oestrogen production and circulation [33,34]. More specifically, oestradiol increases nitric oxide production and decreases oxidative stress, vascular inflammation, and fibrosis, which is favourable for the maintenance of endothelial function and protection against atherosclerosis. However, the protective role of oestrogen is diminished in menopause, with increased autoantibody immune complexes and inflammatory response, which promote plaque formation and rupture [34]. This is also supported in the literature, where post-menopausal females had more vulnerable plaques that are at higher risk of plaque rupture compared to pre-menopausal females [35]. More importantly, females also have a more pronounced pro-inflammatory macrophage phenotype with increased expression of NLRP3 compared to males [36]. Given that colchicine has the ability to suppress the NLRP3 inflammasome, it would be beneficial for future trials to focus on the difference in utility of colchicine in males and females in suppressing the inflammatory pathway. Historical findings have also reported a higher incidence of CAD in males until the age of 70, at which point females overtake to have a higher incidence of CAD instead [37].

A recent meta-analysis looking at major RCTs in the past few years (LoDoCo, COLCOT, COPS, LoDoCo2, CONVINCE, CLEAR) demonstrated a substantial reduction in MACE, MI, ischaemic stroke and recurrent coronary revascularisation in the colchicine group compared to the placebo group. There was no significant difference in adverse events, all-cause and non-cardiovascular mortality. Another meta-analysis (LoDoCo, COLCOT, COPS, LoDoCo2, CONVINCE, CLEAR, CHANCE3, COLOCT, Deftereos et al.) showed similar outcomes—a 12% reduction in cardiovascular death, myocardial infarction or stroke and a non-significant difference in non-cardiovascular death but significant for gastrointestinal events [38]. However, compared to an earlier meta-analysis, it showed a significant difference in non-cardiovascular mortality and no difference in all-cause mortality [39]. Given the heterogeneity in non-cardiovascular mortality, colchicine adverse events, and similar all-cause mortality, it raises a concern regarding the use of colchicine for CAD. While colchicine could improve cardiovascular outcomes, it could come at a risk of non-cardiovascular outcomes and adverse effects while on the treatment.

Recent trials, including COLOCT and EKSTROM, both showed a reduction in minimal fibrous cap thickness and dense calcified plaque volume, respectively, which supports the mechanism of action of colchicine [26,28]. Another downstream effect of colchicine is the reduction in hsCRP. COLCOT and CONVINCE were the only two trials that attempted to evaluate the utility of colchicine in reducing hsCRP. While they did show improvements in hsCRP, both were limited in their generalisability given the small sample size [6,8]. COLCOT and CONVINCE showed an improvement in the colchicine group from 4.27 mg/L to 1.37 mg/L and 2.71 mg/L to 1.52 mg/L, respectively. This is in comparison to 5.09 mg/L to 1.60 mg/L and 2.82 mg/L to 1.91 mg/L, respectively, in the placebo group. The Physicians’ Health Study investigating the prognostic role of hsCRP in primary prevention demonstrated that hsCRP of at least 2.11 mg/L was associated with an increased risk of MI and stroke [40]. The 2019 ACC/AHA guidelines have a Class 2A recommendation for measurement of hsCRP in patients with an intermediate risk for CAD and suggest lipid-lowering therapy for patients with hsCRP of at least 2 mg/L [41]. Extrapolating from this, it is highly likely that a lower hsCRP below 2 mg/L would further reduce inflammation in CAD and improve cardiovascular outcomes. Hence, the lower hsCRP noted with the use of colchicine also suggests a favourable anti-inflammatory effect, which may contribute to its observed benefit in reducing MACE.

The results from the CLEAR-SYNERGY trial were discordant from all other trials discussed. However, a key limitation which cannot be dismissed was that recruitment of the majority of patients occurred during the COVID-19 pandemic. This significantly affects the validity of the trial’s results given the immense strain healthcare systems faced globally during the pandemic. It is vital to note that the pre-pandemic analysis supported the use of colchicine compared to the placebo group. CLEAR-SYNERGY remains the largest RCT looking at the utility of colchicine in the cardiovascular setting despite the less-than-optimal study timing. This result should be taken in a neutral manner until further large-scale RCTs are published.

## 7. Future Directions

Although CLEAR-SYNERGY’s findings are noteworthy, previous positive data suggest clinicians may still consider colchicine for patients with established CVD. Nevertheless, inconsistent trial results and gastrointestinal side effects mean routine use is not recommended.

Chronic heart failure is another condition closely linked with inflammation, which has been demonstrated in the TIME-CHF and RELAX trials [42,43]. Heart failure induces inflammation due to wall stress with a resultant release of cytokines including IL-1β, IL-6, TNF-α—IL-1β and TNF-α promoting ventricular remodelling, endothelial and contractile dysfunction, and IL-6 stimulating myocardial fibrosis and ventricular stiffening, which result in heart failure progression [44,45,46]. Similarly, the NLRP3 inflammasome remains central to the inflammatory cascade in heart failure. NLRP3 activation in endothelial cells is triggered by ischemia, oxidative stress, and cellular injury and leads to downstream release of cytokines, which further amplify local and systemic injury, promoting further myocardial injury [44]. While the CANTOS trial looked at patients post-myocardial infarction, it did show that canakinumab resulted in reduced hospitalisations for heart failure and heart failure-related mortality in patients with known heart failure [4]. Another study investigating the use of anakinra in heart failure patients demonstrated a reduction in hsCRP and N-terminal pro–B-type natriuretic peptide (NT-proBNP) after 12 weeks [47]. These positive results support the targeted use of anti-inflammatories, but more research will need to be done in this field to determine whether colchicine could be the target anti-inflammatory of choice in heart failure or ACS patients. Further insight is expected from the ongoing COLCOT-T2D, COLICA, and COLCARDIO-ACS trials, which focus on colchicine in diabetes, heart failure, and acute coronary syndrome.

## 8. Conclusions

Colchicine has proven effects on atherosclerosis but may not be the universal panacea for CAD as previously thought. There remains a clinical need for colchicine, but its use should be more nuanced with careful weighing of polypharmacy and adverse effects until we have the results from more targeted upcoming trials.

## Figures and Tables

**Table 1:** Overview of colchicine trials.

Trials	Total Number of Patients	Population	Colchicine Dose	Comparator	Follow-Up (Months)	Baseline hsCRP (mg/L)	Primary Outcome
LoDoCo2	5522	CAD	0.5 mg once daily	Placebo	28.6	Data was not collected	CV death, spontaneous MI, ischaemic stroke or ischaemia
COLCOT	4745	MI	0.5 mg once daily	Placebo	22.6	4.27	CV death, resuscitated cardiac arrest, MI, stroke, or urgent hospitalisation for angina requiring revascularisation
COPS	795	ACS	0.5 mg twice daily for 1 month, 0.5 mg once daily after	Placebo	12	Data was not collected	Composite death from any cause, ACS, ischaemia-driven urgent revascularisation and non-cardioembolic ischaemic stroke
CONVINCE	3144	Non-severe ischaemic stroke or high-risk TIA	0.5 mg once daily	Nil colchicine	34	2.71	First recurrent non-fatal ischaemic stroke, MI, cardiac arrest, or hospitalisation
CLEAR-SYNERGY	7062	MI with PCI	0.5 mg twice daily (>70 kg) or 0.5 mg daily (<70 kg) for 90 days and 0.5 mg once daily thereafter	Placebo	36	Data was not collected	CV deaths, recurrent myocardial infarction, stroke or unplanned ischaemia-driven coronary revascularisation
COLOCT	104	ACS with lipid-rich plaque on OCT	0.5 mg once daily	Placebo	12	1.2	Change in minimal fibrous cap thickness
EKSTROM	72	CAD	0.5 mg once daily	Placebo	12	Data was not collected	Rate of change in low attenuation plaque volume as measured by CCTA

Abbreviations: CAD, coronary artery disease; CV, cardiovascular; hsCRP, high sensitivity C-reactive protein; MI, myocardial infarction; OCT, optical coherence tomography; PCI, percutaneous coronary intervention; TIA, transient ischaemic attack.

## Data Availability

The original contributions presented in this study are included in the article. Further inquiries can be directed to the corresponding author.

## Glossary

ACS	Acute Coronary Syndrome
AHA	American Heart Association
CAD	Coronary Artery Disease
CANTOS	Canakinumab Anti-Inflammatory Thrombosis Outcome Study
CI	Confidence Interval
COLCOT	Colchicine Cardiovascular Outcomes Trial
COLOCT	Colchicine–Optical Coherence Tomography Trial
COPS	Colchicine in Patients with Acute Coronary Syndrome Trial
CVD	Cardiovascular Disease
ESC	European Society of Cardiology
HR	Hazard Ratio
hsCRP	High Sensitivity C-Reactive Protein
LDL-C	Low-Density Lipoprotein Cholesterol
LoDoCo2	Low-Dose Colchicine Randomised Controlled Trial
MACE	Major Adverse Cardiovascular Events
NSTEMI	Non-ST Elevation Myocardial Infarction
OR	Odds Ratio
PAV	Percent Atheroma Volume
PCI	Percutaneous Coronary Intervention
RCT	Randomised Controlled Trial
STEMI	ST Elevation Myocardial Infarction
TIA	Transient Ischaemic Attack
